# Ia excitatory postsynaptic potentials are potentiated in slow-type motoneurons after a 5-week treadmill endurance training in rats

**DOI:** 10.1007/s00421-025-06098-1

**Published:** 2025-12-19

**Authors:** M. Krauze, M. Bączyk, H. Drzymała-Celichowska, P. Krutki

**Affiliations:** 1 Department of Neurobiology, Poznan University of Physical Education, 27/39 Królowej Jadwigi St., 61-871 Poznań, Poland; 2 Department of Biochemistry, Poznan University of Physical Education, Poznań, Poland

**Keywords:** Treadmill running, EPSP, Muscle spindles, Synaptic input, Rat

## Abstract

The electrophysiological properties of motoneurons (MNs) are modified in response to motor activity; however, it remains unknown whether adaptations also involve peripheral input from muscle spindles, which are a potent source of excitatory connections to MNs. The aim of this study was to investigate whether endurance training evoked adaptive changes in Ia afferent synaptic transmission to MNs. Male Wistar rats (n = 15) underwent a 5-week treadmill running course. An electrophysiological experiment was performed on each rat under general anesthesia one day after the final training session. A corresponding control group of untrained rats (n = 15) was included. Lumbar spinal MNs innervating the medial gastrocnemius (MG, n = 178) or lateral gastrocnemius and soleus (LG-Sol, n = 186) muscles were investigated intracellularly to record passive membrane properties and parameters of monosynaptic Ia excitatory postsynaptic potentials (EPSPs) evoked from homonymous or heteronymous afferents from synergistic muscles. Between-group comparisons were performed using a generalized linear mixed model. Potentiation of Ia heteronymous EPSPs was observed in slow-type MNs. EPSPs were on average larger by 34% in MG and 44% in LG-Sol MNs; positive correlations of EPSP amplitudes with input resistance were observed. No significant changes were observed for parameters of homonymous EPSPs. The adaptations to endurance training may be attributable to a greater size and/or number of Ia synapses on MNs or altered levels of presynaptic inhibition of Ia fibers. Selective enhancement of synaptic transmission to slow MNs is likely related to the greater contribution of slow motor units during treadmill exercises.

**Graphical abstract:**

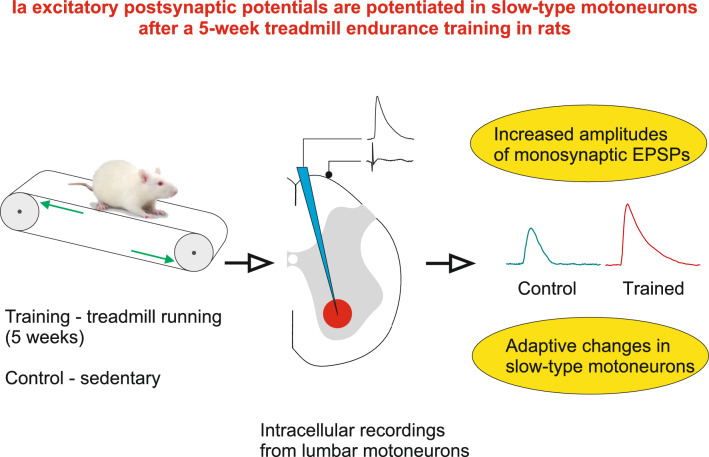

## Introduction

Previous animal and human studies have established that endurance training enhances muscle performance and resistance to fatigue (Vila-Chã et al. 2010; Grassi et al. 2015), and induces pronounced changes in the contractile properties of motor units, even after two weeks of the training (Kryściak et al. 2018). In human studies, both strength training and endurance training enhance the output of the motoneuron (MN) pool, suggesting additional recruitment of motor units during exercise (Vila-Chã et al. 2010), and moderate endurance training has been shown to increase the proportion of recruited low-threshold motor units (Pérot et al. 1991). On the other hand, it has been proven in several studies that endurance training does not affect muscle fiber number or cross-sectional area, but it alters metabolic responses and suppresses hypertrophy (Hendrickse et al. 2021; Shirai et al. 2021).

MNs are also capable of adaptations to this type of increased motor activity; however, due to methodological constraints, only animal models are available for direct experimental studies. A previous study demonstrated that following long-term treadmill endurance exercises, MNs of trained rats had hyperpolarized resting membrane potentials and spike trigger levels, and that fast-type MNs had significantly higher cell electrical capacitance, suggesting increased sizes (Beaumont and Gardiner 2003). However, later morphological studies did not confirm changes in MN size after endurance training (Ishihara et al. 2004). Moreover, rhythmic firing properties were found to be dependent on physical activity level (McDonell et al. 2012).

Surprisingly, it remains unknown whether activity-dependent adaptations also involve peripheral inputs to MNs from muscle receptors, particularly muscle spindles, which have direct monosynaptic connections to MNs and are a potent source of excitatory input to MNs, innervating both homonymous and synergistic muscles (Binder et al. 1993, 2002; Nielsen et al. 2019). Muscle spindles react primarily to muscle stretching, but are also activated during voluntary contractions as they are stimulated by gamma and beta MNs (Banks 1994). Afferent sensory neurons generate action potentials with frequencies that correspond to the size of the stretch and to the rate of stretching (De-Doncker et al. 2003). Moreover, muscle spindles not only signal length changes of the muscle in which they are located but also local length changes that occur as a result of changing the length and relative position of synergistic muscles (Smilde et al. 2016). Therefore, physical exercises are likely to increase the activity of the muscle spindles, as indeed has been suggested in human studies based on the H-reflex response (Pérot et al. 1991). Our recent study in rats revealed that systematic whole-body vibration training, based on extensive activation of muscle spindles, enhanced synaptic transmission from primary muscle afferents in rat spinal MNs (Krutki et al. 2022).

The aim of this study was to determine, for the first time, the effects of 5-week treadmill endurance training on monosynaptic excitatory postsynaptic potentials (EPSPs) evoked in lumbar MNs from Ia afferent fibers originating from muscle spindles. To increase the reliability of the results, we measured the proprioceptive input from two pools of spinal MNs recruited during treadmill locomotion innervating the medial gastrocnemius (MG) or synergistic lateral gastrocnemius and soleus (LG-Sol) muscles, and compared the Ia EPSP parameters of fast and slow MN types. Additionally, in different MNs, we recorded monosynaptic EPSPs evoked by stimulation of the afferents from synergistic muscles (LG-Sol and MG, respectively) and from homonymous Ia afferents from parent muscles. We hypothesized that changes in synaptic transmission to MNs evoked by endurance training are not equally observed in slow and fast types of MNs, due to their likely uneven contribution to motor performance during treadmill exercises.

## Materials and methods

### Animals

Experiments were conducted on 30 adult male Wistar rats (3 months old at the beginning of the study) randomly assigned to the Control (n = 15; body mass 511 ± 63 g) or Endurance (n = 15; body mass 512 ± 67 g) groups. Prior to the experiments, the animals were housed in standard laboratory cages under appropriate conditions (humidity 55 ± 10%; temperature 22 ± 2 °C) with a reverse light/dark cycle (12/12 h) and unrestricted access to food and water.

### Treadmill endurance training

Rats were trained on an electrical treadmill for small rodents (Exer-6 M Treadmill; Columbus Instruments, Ohio, USA). Animals were accustomed to the treadmill during an initial adaptation period of 2 weeks (10–20 min per day at a speed of 10 m min^−1^). Training was conducted 5 days per week, at the same time each day, for 5 weeks. Due to the nocturnal activity of the animals, a reversed day/night cycle was introduced in the animal room, and training took place under weak red light. The schedule was similar to the protocol previously used by our research group (Kryściak et al. 2018; Grzelak et al. 2023). Training intensity was lowest during the first week (20 min. of continuous run at 14–20 m min^−1^; total distance during a session 284–390 m), and increased in subsequent weeks according to the following protocol: second week, 40 min. of continuous run at 16–20 m min^−1^, with 30 s accelerations every 10 min. (distance 632–792 m); third week, 60 min. of continuous run at 16–20 m min^−1^, with 30 s accelerations every 10 min. (distance 942–1200 m); fourth week, 80 min. of continuous run at 17–26 m min^−1^, with accelerations every 10 min. (distance 1392–2080 m); fifth week, 80 min. of continuous run at 21–33 m min^−1^, with accelerations every 10 min. (distance 1640–2616 m). The overall distance covered by individual rats during all 25 training sessions ranged from 24.7 to 32.4 km. The treadmill endurance running regimen used in this study can be classified as moderate to high intensity exercise (Dudley et al. 1982; Cavalcanti et al. 2009; Lalanza et al. 2015).

### Electrophysiological experiment

An electrophysiological experiment was conducted on each rat (one day after the final training session in the Endurance group). The animals were deeply anesthetized with intraperitoneal injections with a cocktail of fentanyl (0.02 mg kg^−1^, Polfa, Poland), midazolam (0.02 mg kg^−1^, Polfa, Poland), and medetomidine (0.4 mg kg^−1^, Cp-Pharma, Poland) at a dose of 10 ml kg^−1^. Supplementary doses of 1 ml kg^−1^ were administered intravenously every 2 h to maintain anesthesia. The depth of anesthesia was controlled by observation of a lack of pinna and withdrawal reflexes during preparation, and continuous heart rate monitoring (300–360 beats per minute) using electrocardiography during recording sessions.

The surgical procedure consisted of the following steps: (1) catheterization of the right jugular and femoral veins for drug administration; (2) endotracheal intubation for artificial ventilation; (3) left-sided preparation of the MG and LG-Sol nerve branches for further electrical stimulation; and (4) laminectomy over the L4–L5 spinal cord segments for placing of recording electrodes. The animals were then placed in a metal frame, positioned on a heated stand, and the vertebral column stabilized with steel clamps. The dissected nerves and exposed area of the spinal cord were then covered with paraffin oil. The animals’ core and oil temperatures were maintained within physiological limits (37° ± 1 °C) using an automatic heating system (Model 507222F, Harvard Apparatus). Subsequently, the dura was removed and small holes were made in the pia to insert glass micropipettes into the spinal cord.

The rats were artificially ventilated during the recording sessions; end-tidal CO₂ level was monitored and maintained between 3 and 4% by adjusting ventilation parameters (Capstar 100, CWE). To paralyze the muscles and enable artificial ventilation during the recording sessions, pancuronium bromide (Pancuronium, Jelfa, Poland) was administered intravenously every 30 min (first dose: 0.4 mg kg^−1^; supplementary doses: 0.2 mg kg^−1^).

At the end of all procedures, rats were euthanized by overdose of pentobarbital sodium (180 mg kg^−1^, Polpharma, Poland). MG, LG, and Sol muscles were collected from all rats and weighed to control effects of the training.

Bipolar silver ball electrodes connected to a square pulse stimulator (DS4; Digitimer, USA) were used for antidromic stimulation of the nerve branches. A silver ball electrode was placed on the dorsal surface of the spinal cord to record afferent volleys from the stimulated nerve. Glass micropipettes with tips 1.5–2.0 µm in external diameter (impedances of 10–15 MΩ) and filled with 2 M potassium citrate were used for intracellular recordings from single MNs located in lumbar spinal cord segments (L4–L5) when EPSPs were evoked from heteronymous, synergistic muscles. A mixture of 2 M potassium citrate with 0.1 M sodium channel blocker QX-314 (Sigma-Aldrich) was used when excitatory EPSPs were evoked from homonymous muscles in order to prevent spiking. Electrodes were inserted into the spinal gray matter using a step motor-driven manipulator with steps of 2–4 µm.

**Fig. 1:**
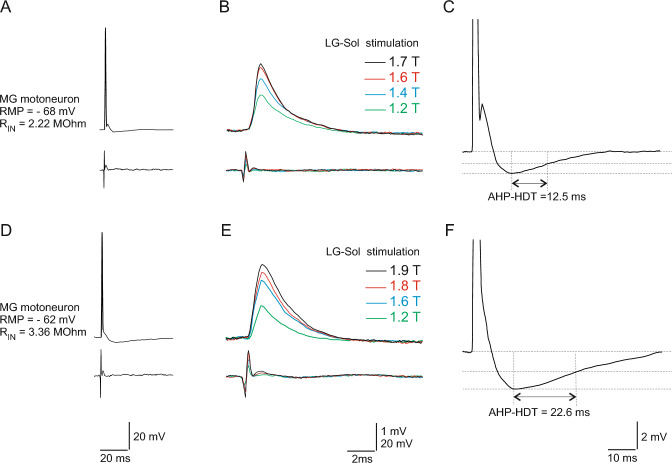
Example recordings from two MG motoneurons (MNs): a fast one (**A**–**C**) and a slow one (**D**–**F**). A and D, antidromic action potentials used for MN identification. B and E, increasing amplitude of monosynaptic excitatory postsynaptic potentials evoked by the stimulation of Ia afferents from synergistic LG-Sol muscles (stimulus intensity expressed in multiples of the threshold for the most excitable fibers in the nerve). Upper traces are intercellular records. Lower traces are incoming volley records from the spinal cord dorsal surface. C and F, the afterhyperpolarization of the orthodromically evoked action potential, indicating difference in its time course between fast and slow MN types. *AHP-HDT* after hyperpolarization half-decay time; *LG-Sol* lateral gastrocnemius and soleus; *MG* medial gastrocnemius; *RIN* input resistance; *RMP* resting membrane potential

Intracellular recordings were acquired with an intracellular amplifier system (Axoclamp, model 2B, Axon Instruments) connected to a Power1401 interface (CED, UK) operated by Spike2 software (CED, UK). The amplifier system was used in bridge mode to record antidromic spikes and EPSPs; discontinuous current clamp mode (switching rate 5.5–8 kHz) was used to record orthodromic action potentials and determine input resistance (R_IN_). A single MN was identified by antidromic stimulation of the nerve to the MG or LG-Sol muscles (Fig. 1 A, D). The antidromic nature of the recorded action potential was recognized on the basis of an all-or-none appearance and a stable, short-latency spike. This method of MN antidromic identification remained possible while using the sodium channel blocker, as it requires 30–90 s for QX-314 to diffuse from the electrode tip to the cell body and block the spike. Only stable recordings with a resting membrane potential (RMP) of at least − 50 mV and action potential amplitudes exceeding 55 mV with clear positive overshoot were considered. Twenty superimposed antidromic action potentials were automatically averaged for further analysis.

The MN R_IN_ was obtained by measuring the membrane voltage deflection in response to a series of short small-amplitude square current pulses (− 1 to + 1 nA, 500 ms; Manuel et al. 2009). During recordings without using QX-314, intracellular depolarization current was later injected into individual MNs to induce an orthodromic action potential. The afterhyperpolarization half-decay time (AHP-HDT) was calculated from the 20 averaged orthodromic spikes. It has been proven to be a reliable tool for dividing rat spinal MNs into fast and slow types (Gardiner 1993). MNs with AHP-HDTs shorter than 20 ms were classified as fast, while those with AHP-HDTs of 20 ms or longer were classified as slow (Fig. 1 C and F).

EPSPs from primary muscle afferents were evoked in MG and LG-Sol MNs by stimulation of afferents from synergistic muscles (LG-Sol and MG, respectively), or afferents from homonymous muscles when QX-314 was used. Constant current stimuli of 0.1 ms duration were delivered at 3 Hz, at an intensity gradually increasing from 1.1 to 2 times the threshold (1.1–2 T) for the most excitable fibers in the nerve, as estimated based on the first component of the incoming afferent volley recorded from the cord dorsum (Fig. 1 B and E). EPSPs were identified as monosynaptic if the central latency measured from the fastest component of the incoming afferent volley was < 1 ms. As EPSPs from group II muscle afferents could be activated > 1.5–1.6 T (Jack 1978), possible overlapping components of disynaptic EPSPs were identified based on noticeable changes in EPSP shape or duration and additional components of incoming volley records; those EPSPs were excluded from further analysis. The amplitude, rise time, half-decay time, and total duration were measured from 40 recordings for the maximum EPSPs (Fig. 1 B and E).

### Statistical analysis

Statistical analyses were programmed in RStudio 2023.06.1 (Posit Software, PBC) with appropriate libraries. A generalized linear mixed model, including zero-inflation and dispersion corrections, was plotted using the glmmTMB package (Brooks et al. 2017) to compare electrophysiological data between the Endurance and Control groups, and to account for the possible dependence of the results on individual animal characteristics (Highlander and Elbasiouny 2022). For MNs in which the EPSPs were evoked from heteronymous afferents, the fixed effects factors were the group (Control, Endurance) and MN type (fast, slow), while rat was the random intercept. For MNs in which EPSPs were evoked from homonymous afferents, the MN type factor was dropped from the model. To validate the fitted model, a Residual Diagnostics for Hierarchical (Multi-Level/Mixed) Regression Model was per-formed using the DHARMa package (Hartig 2024). A plot of the scaled residuals was created by simulating from the fitted model; data deviation, dispersion, and variance were assessed. Where a significant deviation from the model was found, the dispersion or data family correction was introduced and the model was refitted. Only the models that met all assumptions were used for further analysis. The significance of the fixed effect and interaction was established with joint tests of the terms in a model. Between-group significance was then assessed with estimated marginal means for specified factors from the emmeans package (Lenth 2023), with the Tukey adjustment method for comparing a family of estimates. The significance level of the fixed effect was set at α = 0.05. The estimated marginal mean, standard error, and lower and upper limits of the 95% confidence interval are reported for each comparison.

The Pearson’s correlation coefficient (r) and linear regression were used to assess the relation-ships between EPSP amplitude and R_IN_. An equal slopes test was used to compare the slopes of regression lines for these correlations. R_IN_ was compared with an analysis of variance (ANCOVA) between the Control and Endurance groups, controlling for differences in EPSP amplitudes at baseline. Comparisons between the Control and Endurance groups with respect to rat body and muscle mass were performed with a Student’s t-test.

## Results

### Body and muscle mass

No significant differences in rat mean body mass were observed before the electrophysiological experiments (after the training period) between the Control and Endurance groups. The respective values were 511 ± 63 g and 512 ± 67 g (p = 0.489).

The mean MG muscle mass was 1111 ± 140 mg in the Endurance group, which did not differ significantly from the Control group (1145 ± 151 mg; p = 0.261). Similarly, no significant differences in mean LG muscle mass were observed between the Endurance (1274 ± 187 mg) and Control (1274 ± 155 mg; p = 0.261) groups. Sol muscle mass in the Endurance group was also comparable to the Control group (214 ± 43 mg and 235 ± 51 mg, respectively; p = 0.124). Muscle-to-body mass ratios also did not differ between the Endurance and Control groups for the MG (0.217 ± 0.012% and 0.224 ± 0.021%; p = 0.069) and LG (0.248 ± 0.011% and 0.250 ± 0.022%; p = 0.389). However, for the Sol, composed almost exclusively of slow muscle fibers, this ratio was lower in the Endurance than in the Control group (0.041 ± 0.004% and 0.046 ± 0.006%; p = 0.016), indicating a relative decrease in Sol muscle size.

### MN sampling

A total of 364 MNs innervating the muscles substantially activated during locomotion on a treadmill were analyzed in this study, of which 178 were antidromically identified as MG and 186 as LG-Sol. In the Control group, heteronymous EPSPs from synergistic muscles were recorded from 49 MG MNs (30 classified as fast and 19 as slow) and 55 LG-Sol MNs (36 fast and 19 slow). Homonymous EPSPs were recorded from 32 MG MNs and 36 LG-Sol MNs. In the Endurance group, heteronymous EPSPs from synergistic muscles were recorded from 62 MG MNs (45 fast and 17 slow) and 59 LG-Sol MNs (41 fast and 18 slow). Homonymous EPSPs were recorded from 35 MG MNs and 36 LG-Sol MNs (Tables 1 and 2).

**Table 1:** Membrane properties and parameters of monosynaptic EPSPs evoked in MG and LG-Sol MNs by stimulation of the heteronymous Ia muscle afferents from synergists (LG-Sol and MG, respectively)

	MG MNs						LG-Sol MNs					
	Fast			Slow			Fast			Slow		
	Control	Endurance	*p* value	Control	Endurance	*p* value	Control	Endurance	*p* value	Control	Endurance	*p* value
n	30	45		19	17		36	41		19	18	
RMP (mV)	– 62.8 ± 1.27 (– 65.3–– 60.3)	– 62.7 ± 1.03 (– 64.7–– 60.6)	0.999	– 61.0 ± 1.59 (– 64.1–– 57.8)	– 64.5 ± 1.68 (– 67.8–– 61.1)	0.436	– 62.2 ± 1.20 (– 64.5–– 59.8)	– 65.2 ± 1.65 (– 67.5–– 63.0)	0.246	– 62.5 ± 1.65 (– 65.8–– 59.3)	– 62.7 ± 1.70 (– 63.1–– 59.4)	0.999
AHP-HDT (ms)	11.5 ± 0.57 (10.3–12.6)	11.2 ± 0.60 (10.0–12.3)	0.982	22.3 ± 0.75 (20.9–23.8)	23.7 ± 0.72 (22.3–25.1)	0.999	11.3 ± 0.57 (10.2–12.4)	11.8 ± 0.55 (10.7–12.9)	0.905	22.8 ± 0.70 (21.4–24.2)	22.8 ± 0.68 (21.4–24.1)	0.999
R_IN_ (MΩ)	1.92 ± 0.12 (1.68–2.16)	1.91 ± 0.09 (1.72–2.11)	0.999	2.81 ± 0.15 (2.51–3.11)	3.07 ± 0.16 (2.75–3.39)	0.639	1.71 ± 0.14 (1.44–1.98)	1.81 ± 0.13 (1.55–2.08)	0.947	2.69 ± 0.17 (2.35–3.03)	2.98 ± 0.18 (2.63–3.33)	0.614
EPSP central latency (ms)	0.61 ± 0.03 (0.56–0.67)	0.67 ± 0.03 (0.62–0.72)	0.494	0.62 ± 0.04 (0.55–0.69)	0.68 ± 0.04 (0.61–0.75)	0.690	0.60 ± 0.03 (0.54–0.67)	0.60 ± 0.03 (0.54–0.66)	0.999	0.68 ± 0.04 (0.60–0.75)	0.69 ± 0.04 (0.61–0.76)	0.998
EPSP amplitude (mV)	2.37 ± 0.18 (2.02–2.72)	2.14 ± 0.16 (1.83–2.46)	0.775	**2.76 ± 0.23 (2.31–3.21)**	**3.77 ± 0.23 (3.33–4.22)**	**0.011**	2.26 ± 0.20 (1.87–2.65)	2.32 ± 0.19 (1.95–2.70)	0.995	**2.44 ± 0.24 (1.97–2.91)**	**3.39 ± 0.24 (2.91–3.87)**	**0.028**
EPSP rise time (ms)	1.35 ± 0.08 (1.20–1.50)	1.24 ± 0.07 (1.10–1.38)	0.760	1.35 ± 0.09 (1.17–1.53)	1.23 ± 0.09 (1.05–1.42)	0.819	1.26 ± 0.07 (1.12–1.41)	1.20 ± 0.07 (1.06–1.34)	0.918	1.40 ± 0.09 (1.22–1.59)	1.33 ± 0.09 (1.15–1.52)	0.952
EPSP half-decay time (ms)	2.27 ± 0.15 (1.98–2.56)	1.94 ± 0.13 (1.67–2.20)	0.327	2.09 ± 0.19 (1.72 – 2.46)	2.10 ± 0.19 (1.73–2.47)	0.999	2.06 ± 0.16 (1.74–2.39)	2.10 ± 0.16 (1.79–2.41)	0.998	1.97 ± 0.19 (1.59–2.35)	2.25 ± 0.19 (1.87–2.63)	0.728
EPSP duration (ms)	7.44 ± 0.39 (6.66–8.21)	6.51 ± 0.34 (5.84–7.19)	0.270	6.90 ± 0.48 (5.95–7.86)	6.75 ± 0.50 (5.76–7.74)	0.996	7.01 ± 0.56 (6.00–8.19)	7.04 ± 0.54 (6.06–8.19)	0.999	6.37 ± 0.58 (5.33–7.61)	7.48 ± 0.67 (6.28–8.91)	0.582

Values presented as estimated marginal means ± SE, lower and upper limits of the 95% confidence interval (in parentheses), and *p* values derived from a generalized linear mixed model used for comparison between control and trained groups. Ia EPSP parameters were measured using the records with the maximum EPSP amplitudes, evoked at a stimulus intensity of 1.5-2 T. Bold depicts data when the difference between the Control and Endurance groups was statistically significant (p < 0.05). *AHP-HDT*, afterhyperpolarization half-decay time; *EPSP* excitatory postsynaptic potential; *LG-Sol* lateral gastrocnemius and soleus; *MG* medial gastrocnemius; *MN* motoneuron; *n* number of neurons; *R*_*IN*_ input resistance; *RMP* resting membrane potential

**Table 2:** Membrane properties and parameters of monosynaptic EPSPs evoked in MG and LG-S MNs by stimulation of the homonymous Ia muscle afferents

	MG MNs			LG-Sol MNs		
	Control	Endurance	*p* value	Control	Endurance	*p* value
n	32	35		36	36	
RMP (mV)	− 65.0 ± 1.59 (− 68.1–− 61.8)	− 63.4 ± 1.60 (− 66.6–− 60.2)	0.487	− 65.7 ± 1.16 (− 68.0–− 63.4)	− 62.2 ± 1.16 (− 64.5–− 59.9)	0.055
R_IN_ (MΩ)	2.23 ± 0.17 (1.92–2.60)	2.30 ± 0.19 (1.96–2.70)	0.785	2.25 ± 0.18 (1.89–2.61)	2.38 ± 0.19 (2.00–2.75)	0.622
EPSP central latency (ms)	0.69 ± 0.02 (0.63–0.71)	0.65 ± 0.02 (0.61–0.69)	0.414	0.66 ± 0.03 (0.61–0.71)	0.59 ± 0.03 (0.54–0.64)	0.069
EPSP amplitude (mV)	3.26 ± 0.26 (2.74–3.77)	3.25 ± 0.29 (2.67–3.83)	0.986	2.94 ± 0.26 (2.48–3.50)	3.20 ± 0.31 (2.66–3.87)	0.517
EPSP rise time (ms)	1.19 ± 0.05 (1.09–1.29)	1.21 ± 0.05 (1.12–1.31)	0.733	1.18 ± 0.06 (1.06–1.31)	1.16 ± 0.07 (1.04–1.30)	0.867
EPSP half-decay time (ms)	2.10 ± 0.11 (1.87–2.32)	2.1 ± 0.10 (1.86–2.29)	0.895	2.04 ± 0.09 (1.85–2.23)	1.94 ± 0.09 (1.75–2.13)	0.455
EPSP duration (ms)	7.59 ± 0.40 (6.80–8.39)	8.12 ± 0.38 (7.36–8.88)	0.345	7.04 ± 0.36 (6.32–7.76)	7.16 ± 0.36 (6.44–7.88)	0.819

Values presented as estimated marginal means ± SE, lower and upper limits of the 95% confidence interval (in parentheses), and *p* values derived from a generalized linear mixed model used for comparison between control and trained groups. Ia EPSP parameters were measured using the records with the maximum EPSP amplitudes, evoked at a stimulus intensity of 1.5-2 T. *EPSP*, excitatory postsynaptic potential; *LG-Sol*, lateral gastrocnemius and soleus; *MG* medial gastrocnemius; *MN* motoneuron; *n* number of neurons; *R*_*IN*_ input resistance; *RMP* resting membrane potential

### Heteronymous Ia EPSPs

Table 1 shows that no differences in passive membrane properties (RMP and R_IN_) were observed between Control and Endurance groups, both for MG and LG-Sol MNs from which heteronymous EPSPs were recorded. Within each group, slow- and fast-type MNs could be distinguished on the basis of AHP-HDT.

The maximum EPSP amplitudes for individual MNs were obtained between 1.5 and 2 T. The central latencies of EPSPs indicated monosynaptic connections of Ia muscle afferents, and no significant differences were observed between Control and Endurance groups for either MG or LG-Sol MNs of fast and slow types.

Comparison of the EPSP amplitudes evoked in individual MNs by maximal group I stimulation of heteronymous afferents from synergistic muscles showed that they were significantly higher for slow MNs in the Endurance than in the Control group, both for MG MNs (p = 0.011) and LG-Sol MNs (p = 0.028). Such differences in EPSP amplitudes between the Endurance and Control groups were not observed for fast MNs (p = 0.775 and p = 0.995 for MG and LG-Sol MNs, respectively). The EPSP rise time, half-decay time, and total duration did not differ significantly between the Control and Endurance groups; this was the case for both fast and slow MG and LG-Sol MNs (Table 1).

**Fig. 2:**
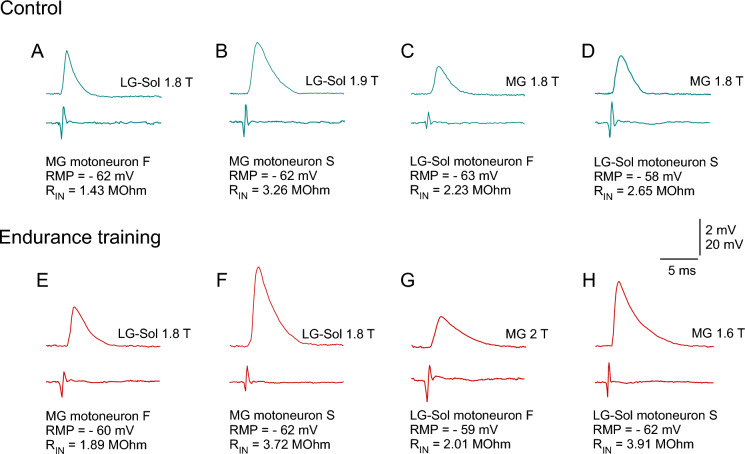
Examples of the maximum Ia excitatory postsynaptic potentials evoked in MG (**A**–**B**, **E**–**F**) or LG-Sol (**C**–**D**, **G**–**H**) motoneurons (MNs) by stimulation of afferents from the respective synergistic muscles in the control (**A**–**D**) and trained (**E**–**H**) groups. Stimulus intensity, expressed in multiples of the threshold (T) of the most excitable fibers, is indicated above each record. MNs were identified as fast (**A**, **C**, **E**, **G**) or slow (**B**, **D**, **F**, **H**); passive membrane properties are indicated below each record. Forty averaged traces for each record. Upper traces are intercellular records. Lower traces are incoming volley records from the spinal cord dorsal surface. *LG-Sol*, lateral gastrocnemius and soleus; *MG* medial gastrocnemius; *R*_*IN*_ input resistance; *RMP* resting membrane potential

**Fig. 3:**
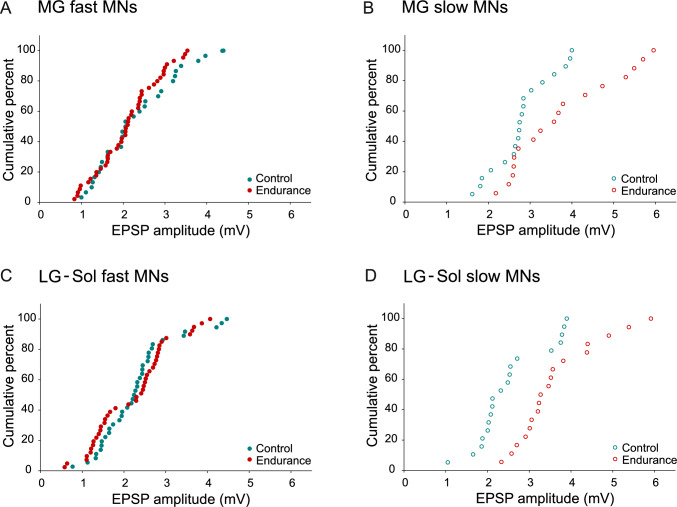
Cumulative histograms showing amplitudes of heteronymous EPSPs across the entire population of MG (**A**, **B**) and LG-Sol (**C**, **D**) motoneurons (MNs). Histograms are plotted separately for fast (**A**, **C**) and slow (**B**, **D**) MNs. Note the rightward shift of the data points for both MG and LG-Sol slow-type MNs of the trained groups (**B**, **D**), for which the mean EPSP amplitudes were significantly higher than in the control group. *EPSP*, excitatory postsynaptic potential; *LG-Sol* lateral gastrocnemius and soleus; *MG* medial gastrocnemius

Figure 2 shows the example recordings for fast and slow MNs of the Control (Fig. 2 A–D) and Endurance (Fig. 2 E–H) groups. Figure 3 presents the distribution of the EPSP amplitudes in cumulative histograms drawn separately for fast and slow MNs. The plots for fast cells are superimposed for both MG and LG-Sol MNs (Fig. 3 A and C). However, there is a rightward shift across the entire pool of data points for EPSP amplitudes recorded from slow MNs of the Endurance group in comparison to the Control; this applies to both MG and LG-Sol MNs (Fig. 3 B and D).

**Fig. 4:**
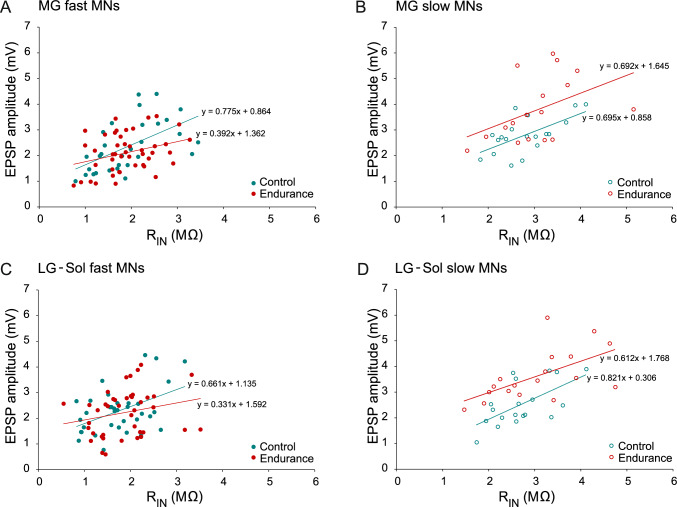
Relationships between RIN and Ia EPSP amplitudes evoked from heteronymous muscle afferents in MG (**A**, **B**) and LG-Sol (**C**, **D**) motoneurons (MNs). Regression lines were determined according to the equations given for each plot. For each pair of correlations, regression line slopes did not differ significantly between the control and trained groups (p = 0.169, p = 0.995, p = 0.302, p = 0.541; equal slopes test for linear regression for **A**, **B**, **C**, and **D**, respectively). Correlations are plotted separately for fast (**A**, **C**) and slow (**B**, **D**) MNs. *EPSP*, excitatory postsynaptic potential; *LG-Sol* lateral gastrocnemius and soleus; *MG* medial gastrocnemius; *R*_*IN*_, input resistance

A linear correlation was observed between EPSP amplitude and R_IN_ (Fig. 4). The respective Pearson’s correlation coefficients for the Control and Endurance groups were as follows: 0.669 (p < 0.001) and 0.312 (p = 0.037) for MG fast MNs (Fig. 4A); 0.627 (p = 0.004) and 0.452 (p = 0.068) for MG slow MNs (Fig. 4B); 0.699 (p < 0.001) and 0.228 (p = 0.152) for LG-Sol fast MNs (Fig. 4C); 0.616 (p = 0.005) and 0.597 (p = 0.009) for LG-Sol slow MNs (Fig. 4D). These associations were not affected by the endurance training, as confirmed by the equal slopes test for linear regression (Fig. 4). The ANCOVA confirmed that Ia EPSP amplitudes for slow MNs in the Endurance group remained significantly larger than in the Control group when the variance arising from R_IN_ was accounted for (F_1, 33_ = 4.011, p = 0.013 for slow MG MNs; F_1, 34_ = 5.9131, p = 0.001 for slow LG-Sol MNs).

### Homonymous Ia EPSPs

Table 2 shows that no differences in passive membrane properties (RMP and R_IN_) were observed between Control and Endurance groups, both for MG and for LG-Sol MNs from which homonymous EPSPs were recorded.

The maximum EPSP amplitudes for individual MNs were between 1.5 and 2 T. The central latencies of EPSPs in this pool of MNs also indicated monosynaptic connections of Ia muscle afferents, and no significant differences were observed between Control and Endurance groups either for MG or LG-Sol MNs. Comparison of the EPSP amplitudes evoked in individual MNs by maximal group I stimulation of homonymous afferents from parent muscles showed no significant differences between the Endurance and Control groups; this was the case for both MG (p = 0.986) and LG-Sol MNs (p = 0.517). The EPSP rise time, half-decay time, and total duration also did not differ between the Control and Endurance groups, for both MG and LG-Sol MNs (Table 2).

**Fig. 5:**
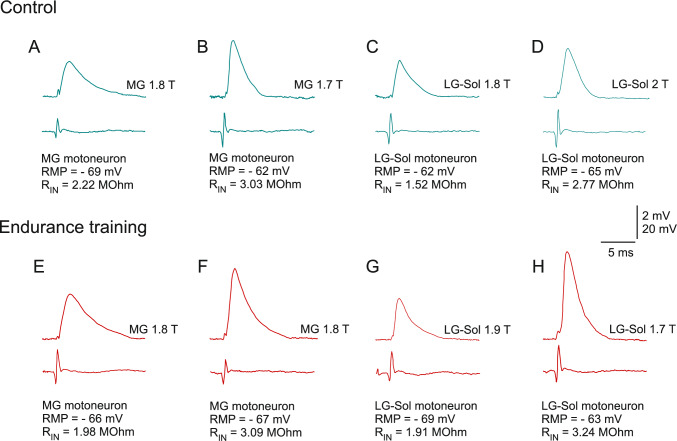
Examples of the maximum Ia excitatory postsynaptic potentials evoked in MG (A–B, E–F) or LG-Sol (C–D, G–H) moto-neurons (MNs) by stimulation of afferents from the homonymous muscles in the control (**A**–**D**) and trained (**E**–**H**) groups. Stimulus intensity, expressed in multiples of the threshold (T) of the most excitable fibers, is indicated above each record. MNs could not be identified as fast or slow as spikes were blocked by QX-314, but passive membrane properties are indicated below each record. Forty averaged traces for each record. Upper traces are intercellular records. Lower traces are incoming volley records from the spinal cord dorsal surface. *LG-Sol*, lateral gastrocnemius and soleus; *MG* medial gastrocnemius; *R*_*IN*_ input resistance; *RMP* resting membrane potential

**Fig. 6:**
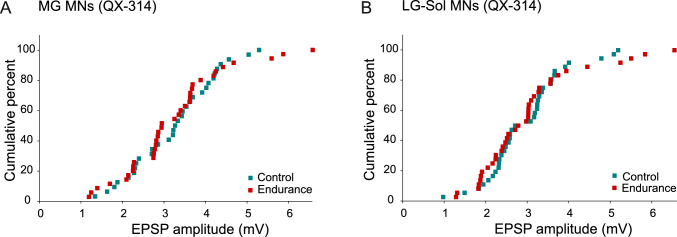
Cumulative histograms showing amplitudes of homonymous EPSPs across the entire population of MG (**A**) and LG-Sol (**B**) motoneurons (MNs). *EPSP* excitatory postsynaptic potential; *LG-Sol* lateral gastrocnemius and soleus; *MG* medial gastrocnemius

**Fig. 7:**
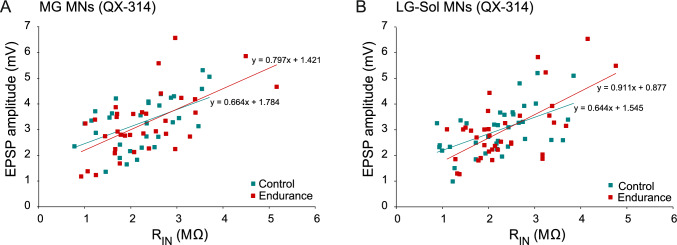
Relationships between RIN and Ia EPSP amplitudes evoked from homonymous muscle afferents in MG (**A**) motoneurons (MNs) and LG-Sol (**B**) MNs. Regression lines were determined according to the equations given for each plot. For each pair of correlations, regression line slopes did not differ significantly between the control and trained groups (p = 0.629 and p = 0.298; equal slopes test for linear regression for A and B, respectively). *EPSP* excitatory postsynaptic potential; *LG-Sol* lateral gastrocnemius and soleus; *MG* medial gastrocnemius; *R*_*IN*_ input resistance

Figure 5 shows the example recordings for MNs of the Control (Fig. 5A–D) and Endurance (Fig. 5E–H) groups. As spike generation was blocked by QX-314, the division into fast and slow types was unreliable in these MNs. However, the presented examples show that MNs with larger EPSP amplitudes had higher R_IN_ values, which is characteristic for slow MNs. Figure 6 presents the distribution of the EPSP amplitudes in cumulative histograms. The plots are superimposed for both MG and LG-Sol MNs. Notably, the largest EPSP amplitudes in the pool were recorded from three MG and three LG-Sol MNs of the Endurance group which also had relatively high R_IN_ values (2.60–4.49 MΩ and 3.07–4.76 MΩ, respectively; see also the distribution of data points in Fig. 7), suggesting they were of the slow type.

Similar to heteronymous EPSPs, a linear correlation was observed between homonymous EPSP amplitude and R_IN_ (Fig. 7). The Pearson’s correlation coefficients were 0.520 (p = 0.002) and 0.609 (p < 0.001) for MG MNs of the Control and Endurance groups, respectively (Fig. 7A), and 0.554 (p < 0.001) and 0.634 (p < 0.001) for LG-Sol MNs of the Control and Endurance groups, respectively (Fig. 7B). These relationships were not affected by the endurance training, as confirmed by the equal slopes test for linear regression (Fig. 7).

## Discussion

The present study demonstrates, for the first time, enhancement of the Ia monosynaptic input from muscle spindles to slow-type spinal MNs in response to 5 weeks of treadmill endurance training. This confirms numerous previous demonstrations of MN plasticity in response to altered muscular activity levels, and complements the knowledge on adaptive changes in MN membrane and firing properties after various forms of running exercises (Beaumont and Gardiner 2003; Gardiner et al. 2006; McDonnell et al. 2012).

### Differentiated changes in Ia synaptic transmission in fast and slow MNs

A growing body of evidence suggests that signals from muscles, as neurotrophins, BDNF or GDNF, retrogradely transported to MNs, are associated with MN function and plasticity, including regulation of excitability and adaptive changes in membrane and firing properties due to altered physical activity (Yan et al. 1993; Copray and Brouwer 1994; Funakoshi et al. 1995; Gonzalez and Collins 1997; Gardiner et al. 2005; Gyorkos et al. 2014). Another study reported that transcriptional changes occur in MNs in response to increases in motor activity, which include receptors and ion channels that could modify motoneuronal responsiveness to input, and thus influence neuromuscular performance (Woodrow et al. 2013). Slow motor units (MNs and muscle fibers innervated by them) are believed to be highly involved in standing, walking and running at low and moderate speed; the least excitable fast motor units are recruited during galloping and vertical jumping (Walmsley et al. 1978). According to Cohen and Gans (1975), adult male rats gallop at a speed ranging from 48 to 75.5 m min^−1^. In this study rats were running on a treadmill at 14–33 m min^−1^, therefore, slow muscle fibers in active muscles are likely a principal source of retrograde signals to spinal MNs during endurance training.

The above considerations are supported by the observation that Sol muscle-to-body mass ratio decreased in the Endurance group. The inverse relationship between muscle fiber size and oxidative capacity is a consequence of oxygen, ADP, and ATP diffusion limitations which constrain fiber size (van Wessel et al. 2010; Hendrickse et al. 2021). Significant changes in the size of MG and LG muscles after training were not detected in this study, likely due to the lower proportion of slow fibers in these muscles. Previous studies on rats indicate that 8–12% of motor units are slow in MG muscle (Celichowski and Drzymała 2006), with 9% in LG muscle (Gillespie et al. 1987). Conversely, Sol muscle is composed predominantly (80–90%) of slow motor units (Close 1967; Gillespie et al. 1987) innervated by slow MNs; however, Sol muscle is much smaller and innervated by a considerably lower number of MNs (Close 1967; Mierzejewska-Krzyżowska et al. 2014).

Selectivity in the adaptive changes in Ia transmission was evident when EPSPs from synergistic muscle afferents were analyzed separately for fast and slow MNs (Fig. 3) and a division between the two types could be performed with considerable certainty (Gardiner 1993). Due to blocked action potentials by QX-314 when EPSPs from homonymous muscle afferents were recorded, such a division could not be reliably performed. Therefore, only entire pools of MNs innervating MG or LG-Sol muscles could be compared, and no significant differences in Ia synaptic transmission can be explained by the high proportion of fast MNs in the analyzed sample. It has been established that EPSP amplitudes are greater for slow than fast MNs (Beaumont and Gardiner 2003, 2017; Cormery et al. 2005; Krutki et al. 2015) and are correlated to R_IN_. From this perspective, it might be expected that the highest EPSP amplitudes, recorded in MNs with relatively high R_IN_ values, would be from slow MNs. This was indeed the case, as the distribution of homonymous EPSP amplitudes in cumulative histograms (Fig. 6) shows that the points with the highest values represent the Endurance group. This applies to both MG and LG-Sol MNs, which is in line with observations of heteronymous EPSPs in which fast and slow MNs could be distinguished.

### Possible mechanisms of increased excitation of MNs

Primary afferents from muscle spindles form Ia synapses with MNs within the motor pool of homonymous and synergistic muscles, enabling widespread excitation of MNs (Eccles et al. 1957; Webb and Cope 1992). Fast MNs within the motor nucleus in the spinal cord receive more Ia contacts than slow MNs (Burke and Glenn 1996). During voluntary activity such as treadmill running, afferents from active muscles provide a net excitation to MNs, reflexively increasing the motor output (Binder et al. 1993; Macefield et al. 1993). The efficiency of this excitation depends not only on the number, size, and functional characteristics of the Ia synapses, but also on the passive membrane properties of MNs, such as RMP and R_IN_ (Heckman and Binder 1988). This study found a significant increase in EPSP amplitudes in slow MNs following treadmill endurance training despite that there were no alterations in RMP or R_IN_; however, EPSP amplitudes in all analyzed groups of MNs demonstrated strong positive correlations with R_IN_ (Figs. 4 and 7), in line with numerous previous studies (Lev-Tov et al. 1983; Carp 1993; Krutki et al. 2022, 2023). The ANCOVA confirmed that EPSPs in slow MNs had significantly higher amplitudes when the variance arising from the R_IN_ was accounted for, indicating that possible training-evoked modulations of the intrinsic properties of MNs were not the principal explanation of the observed adaptive changes in synaptic transmission.

Effectiveness of Ia afferent connections to MNs is also controlled by last-order inhibitory interneurons synapsing directly with the intraspinal terminals of the afferent fibers (Jankowska 1992). A study in cats demonstrated that across the triceps surae population of MNs, the magnitude of presynaptic inhibition by axons from the periphery was correlated with EPSP amplitude, and increased from fast to slow MNs (Zengel et al. 1983). Notably, presynaptic inhibition is facilitated in motor tasks requiring high-level skills, contrary to its inhibition by more automated tasks (Tahayori and Koceja 2012). As treadmill running is primarily an automated locomotor activity, it may be expected that reduced presynaptic inhibition of slow MNs during the exercise facilitated their activation by Ia afferents. This could trigger adaptive responses in the Ia synaptic coverage of spinal MNs, or induce neuroplasticity at the Ia synapse.

Persistent inward currents (PICs) are another possible mechanism contributing to EPSP modulations. While slow calcium PICs have a time course of several hundred miliseconds (Lee and Heckman 1996), the dynamic component of unitary Ia EPSPs can be potentiated by fast sodium PICs (Manuel et al. 2007). However, it remains to be investigated whether PICs can be modulated in MNs in response to endurance training.

Nevertheless, the possibility of changes in Ia synapses formed on MNs cannot be ruled out, as primary afferents and their central connections are capable of significant modifications under both physiological and pathological conditions (Mendell 1984; Koerber et al. 1994). Alterations in the size and number of Ia synapses on spinal MNs due to more frequent or reduced activation of Ia fibers are reported in several studies. For instance, 7-day electrical stimulation of low-threshold afferents was sufficient to increase glutamatergic synapse density on the soma and proximal dendrites of lumbar MNs (Gajewska-Woźniak et al. 2016), while peripheral nerve injury resulted in a significant reduction of Ia synapses on MNs (Rottermann et al. 2014; Schultz et al. 2017). A detailed morphological study is necessary to elucidate the possibility of axonal sprouting, distribution of synaptic boutons, or changes in density of postsynaptic receptors on MNs following long-term endurance training.

### Functional considerations

Several studies have shown that the electrophysiological properties of MNs respond to the chronic activity state, and that the direction and extent of adaptations depend on multiple factors, such as the duration of additional physical activity/inactivity and the type of exercise (Ishihara et al. 2004; Cormery et al. 2005; Gardiner 2006; Krutki et al. 2015, 2017). For instance, 4 weeks of moderate endurance training in rats led to reduced excitability of fast MNs, with no changes in slow MNs detected; this was likely due to less frequent recruitment of the less excitable fast MNs, rather than extensive contribution of slow MNs to muscle activity (Grzelak et al. 2023). In the present study, the training was longer and more intensive, which could more strongly engage slow MNs, in which changes in synaptic input were observed. However, adaptive changes in properties of both fast and slow MN types were observed in rats following 16 weeks of high-intensity running exercises (Beaumont and Gardiner 2003; MacDonell et al. 2012),which could be linked to the effect of superimposed motor activity engaging fast motor units at higher running speeds.

The additional influence of repetitive activation of homonymous and synergistic MNs through feedback reflex loops from contracting muscles during endurance training may also play a role in controlling synaptic function (Koerber and Mendell 1991). However, it remains an open question how efficiently the observed changes in Ia synapses contribute to recruitment of slow MNs and maintaining discharges during activity of slow motor units, particularly as Ia synapses only represent about 2% of all synapses on the MN surface (Burke and Glenn 1996; Fyffe 2001). Nevertheless, studies based on cross-correlograms demonstrated that the probability of MN firing is proportional to the amplitude of both single-fiber and compound EPSPs (Fetz and Gustafsson 1983; Cope et al. 1987). Therefore, it is probable that the observed larger EPSPs in slow MNs after endurance training reflect activity-dependent control of synaptic function and noticeably influence muscular performance.

As in vivo intracellular recordings from human spinal MNs are not possible, these findings in rats cannot be directly verified in humans; therefore, interpretations of certain details should be made with caution. The results of this study may, however, contribute to the understanding of neuromuscular adaptations to endurance training and serve as a source of plausible explanations for training-evoked adaptations in afferent transmission to MNs. Although no consistent pattern of relationships in EPSP properties between different species (mice, rats, cats, and primates) is apparent and the EPSP characteristics of each species are likely unique (Carp 1993; Bączyk et al. 2020; Krutki et al. 2023), general principles of organization of reflex pathways from proprioceptors, including muscle spindle afferent connections in the spinal cord, are comparable across mammals (Jankowska 1992).

Currently MN adaptations or changes in motor unit discharge behavior following endurance training in human subjects can only be assessed indirectly from reflex studies, and their interpretation is limited since they are modulated by several spinal and supraspinal mechanisms. Nevertheless, a higher sensitivity to the mechanical stimuli during reflex activity and increased excitability of the MN pool have been shown in endurance-trained athletes, suggesting recruitment of a larger proportion of low-threshold motor units (Pérot et al. 1991; Kyröläinen and Komi 1994; Maffiuletti et al. 2001). This is consistent with our observations of a potentiated synaptic excitation of slow MNs. Assuming that long-term preferential use of selected muscles can be viewed as a moderate form of exercise, Adam et al. (1998) have reported lower firing rates and lower recruitment thresholds in the dominant hand, which is consistent with the notion of an increased percentage of slow twitch fibers in the preferentially used muscle, allowing twitch fusion and force buildup to occur at lower firing rates. On the other hand, it has been determined in human motor units that the transformation of synaptic input by each MN is a non-linear process and a MN generates output oscillatory components not necessarily present at the input (Farina et al. 2014). Therefore, it remains an open question to what extent changes in synaptic input to MNs from peripheral sources (as shown in our study) influence the motor unit recruitment and firing activity.

### Limitations

There are a few methodological issues to consider. Firstly, the EPSPs were tested at stimulation intensities up to 2 T, which is sufficient to activate all primary afferents in the stimulated nerve, including Ib afferents providing disynaptic inhibitory input to spinal MNs, which could have affected the measured EPSP amplitudes. Inhibitory components were sporadically found on intracellular recordings of postsynaptic potentials, especially at higher stimulus intensities from homonymous afferents influencing the Ia EPSP decay phase. Ib inhibitory input was present in both the Control and Endurance groups (for both MG and LG-Sol MNs), and it was impossible to prevent Ib afferent excitation during electrical stimulation of the nerve branches. However, no differences were found in EPSP half-decay time or total EPSP duration between compared MN pools, indicating that Ib inhibition equally influenced results in all analyzed groups of MNs.

Secondly, the maximum EPSP amplitudes were usually observed at stimulus intensities above 1.5 T, which is often sufficient to activate group II muscle afferents. In this case, however, the group II components, which form disynaptic connections with MNs, could be distinguished on a descending phase of EPSP records; these records were excluded from further analysis. It was possible to reliably measure EPSP time parameters in these MNs at lower stimulation intensities.

Finally, when the study is performed in the anesthetized preparation, MN properties are measured under reduced synaptic influences, while MNs in the awake state of an animal are under neuromodulatory control. Thus, one cannot rule out that overall neuronal activity might be different in awake rats. However, both Control and Endurance groups were compared under the same experimental conditions, and it has previously been reported that monosynaptic spinal reflexes are not reduced by the anesthetics used in this study (Wang et al. 1992; Saberfard et al. 2022).

## Conclusions

The synaptic excitation of rat spinal MNs by Ia afferents was potentiated in response to endurance training in slow but not in fast-type MNs innervating MG and LG-Sol muscles, which acted as plantar flexors during treadmill running. The study confirmed that adaptive responses to increased activation of spinal MNs are relatively quick, being observable after 5 weeks of systematic training. The potentiation of Ia excitation of spinal MNs was not accompanied by alterations in their passive membrane properties, pointing to synaptic plasticity.

## Data availability statement

The dataset that supports the findings of this study can be accessed on a public access repository repod.icm.edu.pl (10.18150/359JLB). All raw recordings are stored in a private data repository at https://box.pionier.net.pl/ provided by Poznan Supercomputing and Networking Center affiliated with the Institute of Bioorganic Chemistry of the Polish Academy of Sciences. Access to the raw data is available upon reasonable request.

## Abbreviations

AHP-HDT: Afterhyperpolarization half-decay time
ANCOVA: Analysis of covariance
BDNF: Brain-derived neurotrophic factor
EPSP: Excitatory postsynaptic potential
GDNF: Glial cell line-derived neurotrophic factor
LG: Lateral gastrocnemius
LG-Sol: Lateral gastrocnemius and Soleus
MG: Medial gastrocnemius
MN: Motoneuron
N: Number
PIC: Persistent inward current
R_IN_: Input resistance
RMP: Resting membrane potential
Sol: Soleus
T: Threshold
